# Nanocarrier System: State-of-the-Art in Oral Delivery of Astaxanthin

**DOI:** 10.3390/antiox11091676

**Published:** 2022-08-27

**Authors:** Nur Rafiqah Abdol Wahab, Meor Mohd Redzuan Meor Mohd Affandi, Sharida Fakurazi, Ekram Alias, Haniza Hassan

**Affiliations:** 1 Department of Human Anatomy, Faculty of Medicine and Health Sciences, Universiti Putra Malaysia (UPM), Serdang 43400, Malaysia; 2 School of Pharmacy, Puncak Alam Campus, Universiti Teknologi MARA (UiTM), Bandar Puncak Alam, Shah Alam 42300, Malaysia; 3 Department of Biochemistry, Faculty of Medicine, Universiti Kebangsaan Malaysia (UKM), Jalan Yaakob Latiff, Bandar Tun Razak, Kuala Lumpur 56000, Malaysia

**Keywords:** astaxanthin, carotenoids, antioxidants, reactive oxygen species, oral delivery, bioavailability, nanoparticles

## Abstract

Astaxanthin (3,3′-dihydroxy-4,4′-diketo-β-β carotene), which belongs to the xanthophyll class, has shown potential biological activity in in vitro and in vivo models including as a potent antioxidant, anti-lipid peroxidation and cardiovascular disease prevention agent. It is mainly extracted from an alga, *Haematococcus pluvialis*. As a highly lipid-soluble carotenoid, astaxanthin has been shown to have poor oral bioavailability, which limits its clinical applications. Recently, there have been several suggestions and the development of various types of nano-formulation, loaded with astaxanthin to enhance their bioavailability. The employment of nanoemulsions, liposomes, solid lipid nanoparticles, chitosan-based and PLGA-based nanoparticles as delivery vehicles of astaxanthin for nutritional supplementation purposes has proven a higher oral bioavailability of astaxanthin. In this review, we highlight the pharmacological properties, pharmacokinetics profiles and current developments of the nano-formulations of astaxanthin for its oral delivery that are believed to be beneficial for future applications. The limitations and future recommendations are also discussed in this review.

## 1. Introduction

As one of the oldest carotenoids discovered from lobster, *Astacus Gammarus*, astaxanthin with chemical formula of 3,3′-dihydroxy-4,4′-diketo-β-β carotene belongs to the class of xanthophyll. Upon discovery, it was only thought to be useful for pigmentation in the aquaculture industry. Interestingly, in 1991, astaxanthin was identified to be a potent antioxidant and the demand for having astaxanthin as a food supplement has been growing each year, worldwide. Currently, it is used in various industries such as food, cosmetics and pharmaceuticals for its orange-red color pigmentation [1,2,3].

Astaxanthin is a metabolic by-product that can also be found in many aquatic animals, for example, salmonids, shrimp and crayfish, which also acts as ultraviolet (UV) light protection, supports immunity response and protection from the oxidation of macromolecules in these organisms [1]. Apart from these aquatic animals, *H. pluvialis* microalgae was recently discovered to accumulate the highest capacity of astaxanthin, reaching up to 9.2 mg/g cell; it is the greatest natural source of astaxanthin for human consumption, to date [2,4]. Concurrently, most of the on-the-shelves astaxanthin is produced synthetically using petrochemicals since the source and method are more cost-efficient for mass production [1]. However, there are arising concerns on the safety and toxicity of long-term synthetic astaxanthin consumption, especially its effects on human health [5]. According to a report by Dong and colleagues, synthetic astaxanthin was banned by the United States Food and Drug Administration (USFDA) for its application in the health food market due to several safety reasons [6]. Nevertheless, this synthetic astaxanthin is still permitted to be used only for certain industries, i.e., the fisheries industry as food colorant in fish feed [4,5]. 

**Figure 1:**
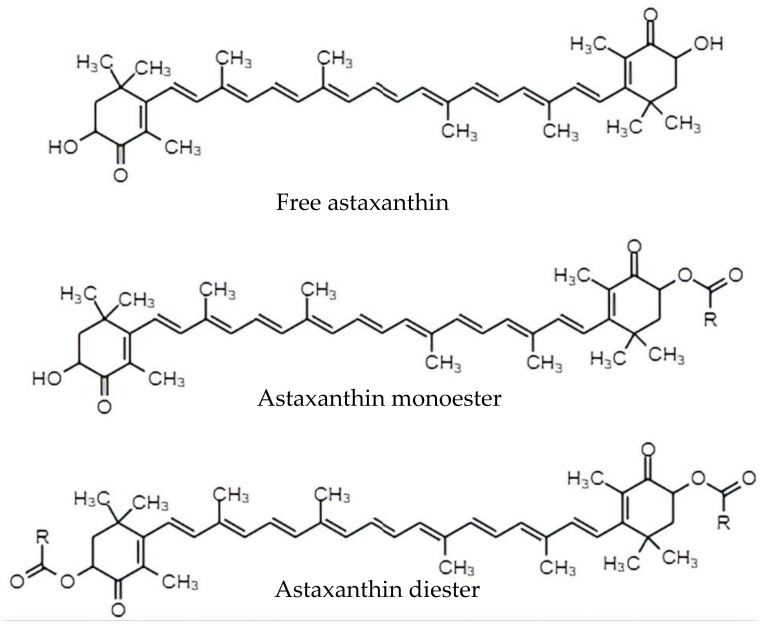
Free form, monoester and diester molecular structure of astaxanthin.

The 100% naturally derived astaxanthin, especially from *H. pluvialis*, is the ‘top pick’ for health application as well as dietary supplementation in humans [1,6]. Natural astaxanthin from the encysted cells of *Haematococcus* can be extracted through various methods which include dissolving in acid before mixing it into either solvents, edible oils or supercritical fluids. For the acid and solvents extraction method, hydrochloric acid will be used for the pre-treatment, followed by extracting the astaxanthin using acetone. The hydrochloric acid recovers almost 80% of the pigment. Then, the encysted cells will be treated using 40% acetone for 2 min at 80 °C followed by dissolving in various solvents such as kitalase, cellulose and acetone powder. This will result in 70% recovery of the astaxanthin. In *Phaffia rhodozyma* yeast for instance, the astaxanthin can be extracted up to 1.3 mg/g cell under acidic conditions. Vegetable oils such as soybean, corn, olive and grapeseed can also be used to extract astaxanthin as this compound is lipophilic. The extraction method involves mixing the culture with the respective oils, where the highest recovery of astaxanthin can be obtained with olive oil with a recovery percentage of 93%. Lastly, the alternative method of extracting astaxanthin is by using supercritical fluid extraction with sunflower oil and ethanol as co-solvents. The yield obtained was in the range of 80–90% by repeatedly extracting astaxanthin with solvents, using a rotary evaporator for evaporation and analyzing the extract using high-pressure liquid chromatography for quantification [4]. Usually, astaxanthin that is extracted from natural sources contains a mixture of monoesters and diesters, while the synthetic astaxanthin is available in its free form as shown in Figure 1 [7]. 

The presence of keto- and hydroxyl groups on each end of astaxanthin molecules, serving as the radical trapping sites, plays an important role in terminating the free radicals chain reaction. This allows it to become a powerful antioxidant with higher bioactivity compared to other carotenoids such as zeaxanthin, lutein and β-carotene [3,8]. Furthermore, the strong antioxidant property of astaxanthin is also contributed by the conjugated polyene chain located at the center that donates the electrons and reacts with free radicals [1,4]. Despite its proven antioxidant activity, this carotenoid was reported to be sensitive towards degradation and its application in the medical and nutraceutical field is still limited. This could also be due to the low solubility and poor bioavailability of astaxanthin in the physiological systems [9]. Therefore, this review intends to discuss the pharmacokinetics and pharmacological properties of astaxanthin and highlight the strategies for antioxidant preservation as well as the enhancement of oral delivery and bioavailability of astaxanthin, particularly the development of potential nanocarrier systems. The recommendations, limitations and prospects for future studies will also be discussed in this review.

## 2. Pharmacodynamics of Astaxanthin

**Figure 2:**
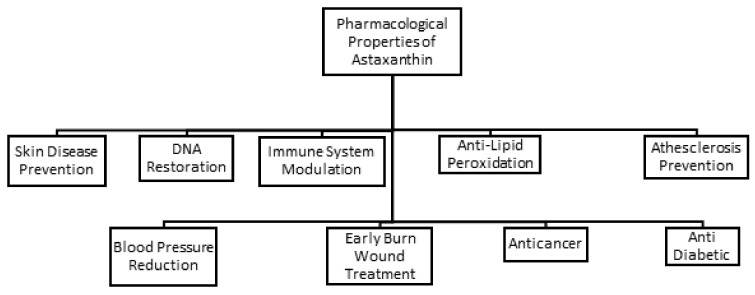
Various pharmacological properties of astaxanthin.

In recent years, astaxanthin has captured growing attention from researchers around the globe for its beneficial health effects and positive physiological responses. The antioxidant property of astaxanthin is highly valuable for its future therapeutic use. Numerous studies have been conducted to demonstrate the antioxidant activities of astaxanthin that are believed to be the key role in protection from oxidative damage, early burn wounds, oncogenic mutation, lipid peroxidation, cardiovascular diseases, inflammation, DNA damage, and diabetes as illustrated in Figure 2.

### 2.1. Skin Disease Prevention

In living organisms, oxidative damage may occur when there is presence of free radicals, generation of reactive oxygen species (ROS) and/or UV light exposure. In addition, if the radicals are produced excessively, they can react with proteins, lipids and DNA through a chain reaction, causing the lipid and protein molecules to oxidize and damage the DNA. In order to inhibit the action of these oxidants and minimize their deleterious effects, antioxidants are well recommended to play their vital role in this event. Astaxanthin, for instance, has proven to prevent skin diseases by inhibiting the ROS formation and controlling the excretion of oxidative stress-responsive enzymes such as heme oxygenase-1 (HO-1) [1,4]. 

Skin is composed of epidermis, dermis and subcutaneous tissues, providing an important protection to the body from physiological injury. ROS can cause damage to the skin through the daily UV-rays exposure from the sun [10]. HO-1 enzyme is involved in the regulatory mechanism used to adapt cells against oxidative damage through nuclear factor erythroid 2-related factor (Nrf2), which is a stress-sensitive transcription factor. Astaxanthin could upregulate the expressions of Nrf-2 and HO-1 in the irradiated cells. In a study by Xue et al., immunofluorescence staining assay showed that astaxanthin enhanced the expression of Nrf-2 and HO-1 in the irradiated mice group. The study also suggested that astaxanthin suppressed the generation of ROS by upregulating Nrf-2 transcription factor and HO-1 expression [11]. Furthermore, endogenous antioxidant enzymes such as glutathione peroxidase 1 (GPX1), superoxide dismutase 2 (SOD2) and Nrf2-targeted proteins HO-1 were highly expressed after the administration of astaxanthin. Therefore, ROS-producing enzymes were reported to be reduced upon upregulation of the endogenous antioxidant enzymes by astaxanthin [1,10,11]. 

### 2.2. Early Burn Wound Treatment

Astaxanthin has also been studied for its potential application as burn wound healing protector. Fang and colleagues studied oxidative stress in the stasis zone of the wounds in “comb” burn rat models and found that astaxanthin (20 mg/kg) had alleviated the burn-induced histological changes in the wound. Further testing revealed that astaxanthin attenuated the ROS-induced oxidative stress in the stasis zone of the burn wound via an inhibited lipid peroxidation, an activated nicotinamide adenine dinucleotide phosphate (NADPH)-dependent oxidase system and enhanced the antioxidant enzymes activities. Xanthine oxidase (XO) and the reduced form of nicotinamide adenine dinucleotide phosphate (NADPH) oxidase (NOx) were involved in the generation of ROS resulting from the burn. ROS acts as secondary messengers to immunocytes in the wound healing process. This suggests that astaxanthin can attenuate oxidative stress that can result in reduced histological changes induced by burn [1,12].

### 2.3. DNA Restoration

Astaxanthin also provides protection against biologically harmful UV radiation that may cause oncogenic mutations in the DNA. It was noted that UV exposure could reduce the endogenous antioxidant defense system of fibroblasts due to the presence of excessive amounts of ROS, causing DNA damage [13]. DNA damage can be minimized by increasing the expression of oxidative stress-response enzymes such as superoxide dismutase (SOD) with the help of exogenous antioxidants such as astaxanthin as the ROS scavenger [14]. In a study by Camera and coauthors, they reported that astaxanthin counteracted cellular changes when human dermal fibroblast (HDF) samples were exposed to UVA. Cellular changes such as inducing apoptosis, increasing levels of ROS and decreasing antioxidant enzymes activities were observed from the fibroblast cultures when exposed to UVA and were improved by astaxanthin by balancing the redox status. On top of that, astaxanthin had also shown a superior photoprotective effect when compared with β-carotene and lutein. In an earlier study, rat kidney fibroblasts (NRK) were exposed to UVA-induced oxidative stress, and they found that astaxanthin could restore the alterations caused by the UVA-induced oxidative stress better than β-carotene and lutein. These photoprotective properties of astaxanthin indicated its potential to prevent a secondary carcinoma caused by UV-induced oxidative stress [13,14].

### 2.4. Anticancer Properties

In addition, astaxanthin also exhibits anticancer properties. Carotenoids like astaxanthin can be exploited to upregulate the connexins [4,15], which are essential in enabling the direct gap junction intercellular communication between cells [16]. In tumor or cancer cells, the direct intercellular communication between cells through gap junctions is poor due to an increased number of oxidants that reduce the expression of connexin-43 proteins. This event leads to poor direct intercellular communication between cells through gap junctions [15,17]. Hix and the research team reported that the expression of connexin-43 proteins was upregulated in mouse embryonic fibroblast cell cultures when treated with disodium salt disuccinate astaxanthin derivatives at 10–5 M in 33% EtOH (1:2; ethanol: water formulations) compared to 33% EtOH as the control solvent. The upregulated connexin improved the cell–cell communications through the gap junctions, and hence reduced cell mutagenesis and carcinogenesis [18].

In another study, astaxanthin exhibited protection against cyclophosphamide-induced oxidative stress and DNA damage in mice [19]. Cyclophosphamide is a cytotoxic alkylating agent used in the treatment of various cancers; however, it also exhibits cytotoxicity to normal cells. In a study by Tripathi and Jena, the findings showed that level of malondialdehyde and DNA damage increased, while the level of antioxidants, glutathione and SOD were reduced, in the presence of cyclophosphamide in male Swiss mice. However, after astaxanthin administration, it was discovered that the oxidative biomarkers such as SOD and malondialdehyde were restored, and DNA damages were decreased. Also, the frequency of chromosomal breakage in the bone marrow cells and peripheral blood reticulocytes was reduced. Therefore, astaxanthin was proven to be a promising chemoprotective agent against cyclophosphamide toxicity [19]. 

### 2.5. Immune System Modulator

Apart from that, astaxanthin was suggested to modulate the immune system defense and offer protection against oxidative damage caused by free radicals generated from UV rays and normal aerobic processes. Previously, Lin et al., assessed the immunomodulatory effects of astaxanthin in primary cultured lymphocytes in in vitro and ex vivo models. The findings of this study exhibited an increment in the weight of rat’s spleen supplemented with oral astaxanthin (0.28, 1.4 and 7 mg/kg/day) for 2 weeks, suggesting that spleen is the main site of astaxanthin accumulation. The study also demonstrated that astaxanthin increased proliferation of splenic lymphocytes (*p* < 0.05) in response to the stimulation of Concanavalin A (Con-A) and Lipopolysaccharide (LPS), which indirectly indicate enhanced immune system of the rats [20,21,22]. This might suggest that the cellular and humoral immune functions could be enhanced with the presence of astaxanthin.

### 2.6. Anti-Lipid Peroxidation

**Figure 3:**
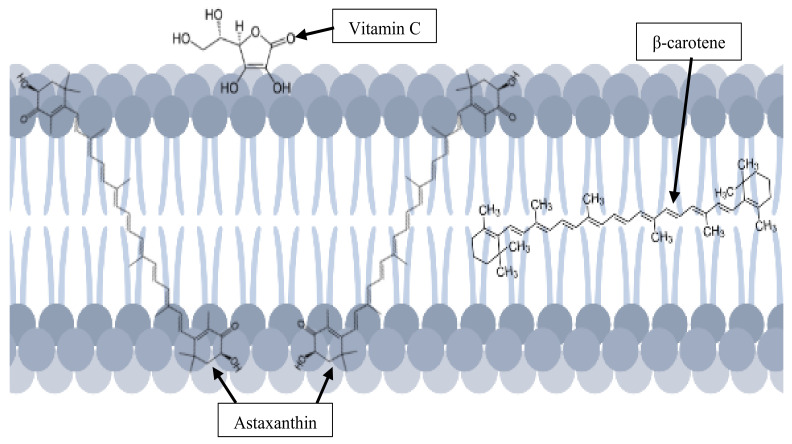
Unique position of astaxanthin in the cell membrane.

In addition, astaxanthin has a unique molecular structure that allows it to be positioned in- and outside of the cell membrane to exert its antioxidant activity against ROS without causing membrane disorder. In contrast to β-carotene and vitamin C, that only stay either inside or outside of the lipid bilayer respectively, astaxanthin has a better position to exert its anti-lipid peroxidation activities, as shown in Figure 3 [4,23]. As a polar xanthophyll, astaxanthin preserves the membrane structure while exhibiting a significant antioxidant activity by reducing the lipid hydroperoxide (LOOH) levels by 40% [24]. It also prevents lipids from becoming oxidized by scavenging on hydrogen peroxide and hydroxyl radicals to terminate the chain reactions [4,8]. There are many pathological conditions caused by lipid peroxidation, which is promoted by ROS production, that consist of free radicals (superoxide, hydroxyl, nitric oxide and many more) and non-radical oxidants (hydrogen peroxide, hypochlorous acid, singlet oxygen and others) [8,25,26].

### 2.7. Atherosclerosis Prevention

Astaxanthin has also been studied thoroughly for its prospective therapeutic uses in the prevention of atherosclerosis [27]. Previously, in vitro and clinical studies were carried out to demonstrate the effect of astaxanthin on low density lipoprotein (LDL) oxidizability by Iwamoto et al. For the in vitro study, it was found that astaxanthin (12.5, 25.0, 50.0 µg/mL) extended the LDL oxidation lag time in the radical initiator assay called V-70 (2,2’-azobis(4-methoxy-2,4-dimethyl-valeronitrile)). It was suggested that the prolonged LDL oxidation lag time indicated an effective ability to reduce the oxidation process. The study was further tested clinically with a total number of 24 volunteers who were supplemented with oral astaxanthin and randomly assigned into five groups: control (*n* = 6), 1.8 (*n* = 5), 3.6 (*n* = 5), 14.4 (*n* = 3) and 21.6 mg (*n* = 5), respectively, to demonstrate the inhibition of LDL oxidation by astaxanthin. The outcomes of this clinical study supported the in vitro finding, which have proven that astaxanthin could inhibit the oxidation of LDL [27]. It was noted that astaxanthin supplementation in the volunteers had prolonged the lag time of LDL oxidation as compared to the other antioxidants such as α-tocopherol and β-carotene. The outcomes of this clinical study supported the in vitro findings, together suggested that astaxanthin could inhibit the oxidation of LDL and could prevent atherosclerosis [27].

### 2.8. Blood Pressure Reduction

With regard to its potential application for the prevention of cardiovascular diseases, astaxanthin was found to lower the blood pressure in spontaneously hypertensive rats (SHR), a well-known model with a significantly high blood pressure along with high oxidative stress [28,29]. After 8 weeks of an astaxanthin-enriched diet (200 mg/kg), Monroy-Ruiz and colleagues found that the systolic blood pressures of the SHR were reduced compared to the control group. Being a potent antioxidant, astaxanthin has also proven to reduce oxidative stress as well as blood pressure in this animal model [29]. The above findings were in good agreement with a previous study by Hussein et al., where they reported that the blood pressure of the stroke-prone SHR (SHR-SP) rats were significantly reduced by 10–25 mmHg when treated with oral astaxanthin (50 mg/kg) for five weeks. Intriguingly, the incidence of strokes was also delayed, with a significant reduction in the arterial blood pressure after the long term administration of astaxanthin [28,29]. The antihypertensive effects of astaxanthin reported from both studies were also associated with the reduction of superoxide anion (·O_2−_), which directly reduces the oxidative stress which also reduces the blood pressure in the animal model. Both studies supported the potential of astaxanthin in reducing blood pressure [28,29].

### 2.9. Anti-Diabetic

Lastly, astaxanthin also possesses an anti-diabetic property and could potentially prevent diabetes. In a study by Uchiyama and others, it was demonstrated that astaxanthin preserved the pancreatic β-cell function in the diabetic mice and improved their glycemic level. The islet cells of the mice treated with astaxanthin (orally mixed in chow 1.0 mg/day) were able to secrete insulin when assessed using the intraperitoneal glucose tolerance test. As a result, the blood glucose levels were significantly reduced, as serum insulin levels improved and glucose toxicity reduced [30]. In a similar study by Zhuge et al., it was found that astaxanthin significantly reduced the blood glucose level of the advanced stage of diabetic rats [31]. After three weeks of astaxanthin treatment, the expression of insulin-related genes such as adiponectin, adiponectin receptor 1 (AdipoR1) and adiponectin receptor 2 (AdipoR2) was increased. These genes are known to have glucose-lowering effects which also ameliorated the insulin resistance in the rats. Diabetes is a disease that is also associated with high levels of oxidative stress, as observed in diabetes mellitus patients [30]. As an antioxidant, astaxanthin has the ability to scavenge radicals that build up to causing oxidative stress, as shown in type 2 diabetes mellitus (T2DM) mice [30,31].

## 3. Pharmacokinetic and Bioavailability of Oral Astaxanthin

Even though astaxanthin has been proposed for various purposes, its applications are significantly limited due to its dissolution hindrances in the gastrointestinal (GI) fluids. It was noted that the oral bioavailability of astaxanthin is relatively low, ranging between 10–50% of a given dose, as a result of its poor solubility in water as well as in lipid blood components such as triglycerides [32]. Consequently, this lipophilic carotenoid is poorly absorbed by epithelial cells in the small intestine and is categorized as a low bioavailability compound [5,33]. The oral bioavailability of astaxanthin varies depending on the time of consumption i.e., before or after a meal as well as the type of lipid-based dietary that is taken along with this carotenoid, and could also be influenced by factors such as smoking [4,34,35].

Upon oral uptake of astaxanthin, this compound follows the absorption of dietary fat due to its lipophilic properties. Its absorption takes place shortly after hydrolysis by cholesterol esterase before it is incorporated into micelles since astaxanthin is usually presented in the esterified forms (mono- and diesters) [32,36]. Despite its low solubility in aqueous environments, astaxanthin can still be absorbed as compared to the other non-polar carotenoids owing to its polar ends in the hydrolyzed form (free form) [36]. Then, the micelles containing astaxanthin are passively absorbed into the intestinal mucosal cells and subsequently incorporated in chylomicrons. Before entering the systemic circulation, chylomicrons containing astaxanthin are released into the lymphatic systems and digested by lipoprotein lipase leaving chylomicron residues to be removed by liver and other tissues. Finally, astaxanthin is absorbed by high-density lipoproteins (HDL) and low-density lipoproteins (LDL) and is then transported to tissue [4,32].

In a study by Osterlie M. and colleagues, it was reported that following oral administration of 100 mg free astaxanthin, the maximum plasma concentration (C_max_) and the time taken to achieve peak plasma concentration (t_max_) were found to be 1.3 ± 0.1 mg/L and 6.7 ± 1.2 hr, respectively. The volume of distribution and oral clearance for free astaxanthin were 0.40 ± 0.2 L/kg and 0.013 ± 0.01 L/hr, respectively with a rate of elimination of 0.042 ± 0.035 hr^−1^ [35]. On the other hand, the oral administration of 100 mg of astaxanthin esters resulted in 0.28 ± 0.12 mg/L maximum plasma concentration with a longer elimination half-life of 52 ± 40 hr^−1^. The volume of distribution and the oral clearance were 2.0 ± 1.3 L/kg and 3.3 ± 1.1 L/hr, respectively with the elimination rate constant of 0.048 ± 0.03 hr^−1^ for the oral diester astaxanthin [37]. Based on these results, it could be presumed that the additional hydrolysis process for the astaxanthin esters has slowed down the rate of astaxanthin absorption.

In a previous study by Okada et al., the oral bioavailability of astaxanthin was observed in 20 subjects at two different timings: before and after a meal. The first group was given 4 mg of astaxanthin in soft capsules 2 h prior to a meal. Meanwhile, the after-meal group was given the same dose of astaxanthin 10 min after having their meal. Each capsule contained 52 mg *Haematococcus* algal extract, olive oil and Vitamin E. They found that the after-meal group exhibited 2.4 times greater area under the curve (AUC)_(0__–168)_, compared to the before-meal group measured until the final time point at 168 h, as shown in Table 1**.** They also noted a significant higher value in the AUC_(0__–∞)_ of the after-meal group than the before-meal group (greater by 2.5 times), suggesting that the absorption of astaxanthin was facilitated by the presence of fat in the after-meal group diet. The fats in the meal stimulated bile excretion that helped in the dispersion of astaxanthin in the digestive system that eventually improved the absorption of astaxanthin [34].

According to a study by Odeberg et al., absorption of astaxanthin was greatly influenced by the lipid-dietary parameters. It appeared that a higher concentration of astaxanthin was absorbed into systemic circulation through oral administration in an oil-based formulation. There were four formulations used in this study, which included one reference formulation consisting of algae meal and dextrin encapsulated in hard-shelled capsules. Three other formulations were differentiated by the addition of lipid components such as long-chain triglyceride (palm oil) for Formulation A, glycerol mono- and dioleate for Formulation B and glycerol mono- and dioleate, and sorbitan monooleate for Formulation C. They revealed that the oral bioavailability of astaxanthin for all formulations was higher compared to the reference by observing the AUC_(0–__∞)_ (Table 1) [32].

Lastly, smoking was also reported to influence the bioavailability of this compound. Okada et al., found that the blood levels of astaxanthin in smokers were lower compared to the non-smokers. The AUC_(0–__∞)_ for non-smoker group was 1.15 times higher than the smoker group. The half-life (t_1/2_) of astaxanthin in the smoker group was shorter, indicating that smoking promoted faster elimination of astaxanthin. Besides, the shorter t_1/2_ of astaxanthin in the smoker group also could be due to the presence of an abundance of free radicals in cigarette smoke that stimulated oxidative stress in the respiratory and circulatory system. Hence, the shorter t_1/2_ of astaxanthin and the greater depletion of astaxanthin as an oxidant in the smoker were the consequences of sustaining the oxidant load caused by smoking. As a result, the blood level of astaxanthin reduced faster in the smoking group [34].

Hitherto, various recommendations have been proposed to enhance the bioavailability of astaxanthin. For example, astaxanthin was suggested to be taken after a meal and/or administered together with a high fat diet apart from changing lifestyle by quitting smoking habits. Among the aforementioned strategies, the application of nano-formulations as potential carriers for astaxanthin is deemed to offer a certainty in enhancing the bioavailability when administering this antioxidant orally. Astaxanthin incorporated into nano-formulations has demonstrated many advantages such as enhanced solubility, reduced degradation, controlled astaxanthin release pattern and improved bioavailability [38].

## 4. Nanocarrier Systems for Oral Delivery of Astaxanthin

Over the years, oral administration of drugs has been the most preferred route for the vast majority of people due to several reasons. Firstly, the oral route is convenient for patients, whereby they can administer it at any time hence, enhancing patient compliance. Also, the manufacturing process of oral dosage form does not require strict sterile conditions, apart from it being less invasive [39]. However, it is quite challenging to achieve effective drug delivery while minimizing the side effects with the conventional oral dosage forms. Therefore, researchers have been putting more effort into designing formulations that can enhance absorption of active pharmaceutical ingredients, especially phytochemicals such as astaxanthin [40]. A nanoparticulate system is one of the oral drug delivery approaches that has been reported to improve drug stability in the gastrointestinal (GI) environment, enhance drug-specific targeting while increasing drug absorption, solubility and bioavailability [41]. Nano-formulations are designed using biocompatible and biodegradable materials in nanoscale size ranging from 10 to 1000 nm. Several types of nano-formulations have been exploited for various purposes such as drug treatment of diseases, to act as selective diagnostic markers, improving the efficacy of novel and old drugs and targeted drug delivery vehicles [42,43].

**Figure 4:**
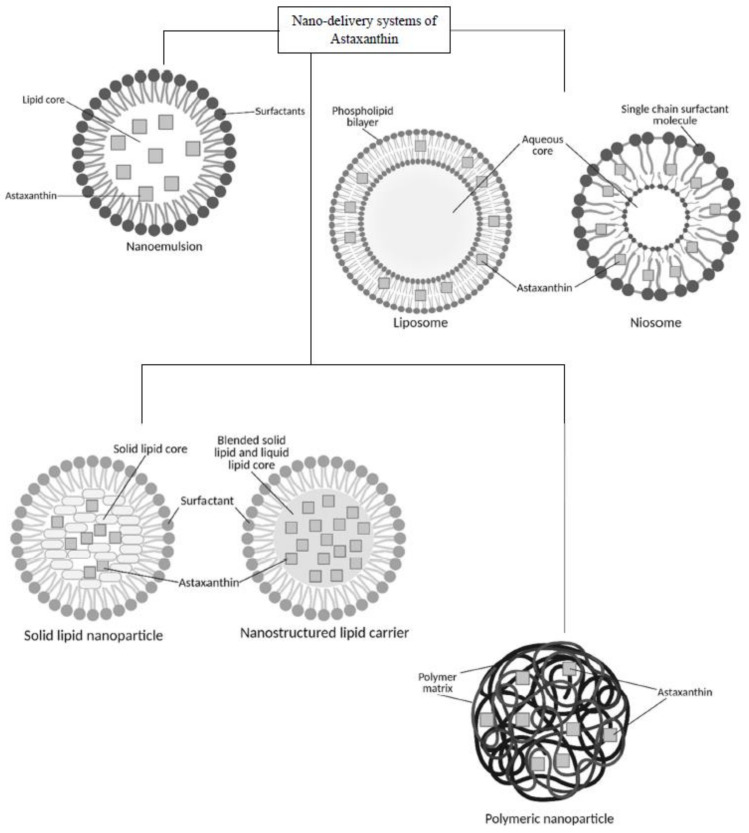
Schematics for nano-delivery systems of astaxanthin.

Nanoparticles as delivery systems are able to minimize the risks of toxicity and adverse effects of many drugs. They have a high surface area to volume ratio, which enables the particles to improve the penetration through cell membranes [5,39,44,45]. Several strategies involving nano-formulations have been employed in astaxanthin delivery research to overcome its pharmacological drawbacks such as low bioavailability and instability [9]. Lipid-based nanoparticles that include nanoemulsions [46], nanostructured lipid carriers [9], niosomes [40] and other polymeric nanoparticles such as chitosan-based carriers [5] were among the formulations developed in the last decade (Figure 4**).** The method of preparation, type of application and its functionality will be discussed thoroughly in this section. Various findings of nanoformulations loaded with astaxanthin from previous studies are summarized in Table 2.

### 4.1. Lipid-Based Nanocarriers—Nanoemulsions, Liposomes, Solid-Lipid Nanoparticles and Nanostructured Lipid Carriers

Lipid-based nanocarriers are primarily made up of lipids, as the name suggested, and this formulation is mostly indicated for the delivery of fatty compounds such as astaxanthin [40]. Lipid components, either liquid or solid lipids such as palm oil, triglyceride and waxes, have been used in the nanoparticles preparation because of biodegradability, non-toxic and hold the generally-recognized-as-safe (GRAS) status [38].

Several of the advantages of formulating astaxanthin into lipid-based nanoparticles include improved bioavailability and having a higher kinetic release. This is due to the metabolic pathway of lipid-based nanocarriers being similar to dietary fats and astaxanthin released from the carriers by simple diffusion [40,47]. Other than that, the chemical compatibility between lipid-based nanocarrier and astaxanthin offers better stability to the formulation [40]. Last but not least, lipid-based nanocarriers exhibited low toxicity due to the lipid components that are biodegradable [39,41].

#### 4.1.1. Nanoemulsions

Nanoemulsions are colloidal particulate systems with small sized droplet emulsions ranging between 10 to 200 nm that are stable against sedimentation. They are produced using oil and an aqueous phase that have been stabilized in the presence of an emulsifier. The emulsifying agent acts as intermediate or interphase to enhance the stability of the emulsion besides the compositions of emulsions and their droplet size [48,49,50].

Nanoemulsions can be formed into three types: (1) water in oil nanoemulsion in which the water droplets are dispersed in the continuous oil phase; (2) oil in water nanoemulsions in which oil droplets are the dispersed phase and water as the continuous phase; (3) bi-continuous nanoemulsions [50]. These nanoemulsions can be synthesized by employing several methods which are categorized as high-energy methods and low-energy methods. The high-energy methods utilize strong disruptive forces producing high kinetic energy that fragments large particles into nano size droplets. Several examples for high-energy methods are high-pressure homogenization, ultrasonication and high-speed homogenization. On the other hand, low-energy methods include phase inversion emulsification method and spontaneous emulsification. Internal chemical reactions are involved in the low-energy method and only requires gentle stirring to produce nanoemulsions [51,52].

One of the approaches that has been explored and demonstrated improved pharmacokinetic properties of astaxanthin is incorporation of this antioxidant into fine particles of oil-in-water nanoemulsions. A recent study discovered that the permeability of astaxanthin loaded into TAP-nanoemulsion (tocopheryl polyethylene glycol succinate, astaxanthin in edible peanut oil) through human colon cancer cell lines (Caco-2) was increased by 80% in a simulated intestinal environment [53]. Caco-2 was employed in this study to resemble the human intestinal absorption of drugs and bioactive compounds, since the small intestine was proposed to be the site of absorption for astaxanthin. These findings suggested that the smaller particle size resulted in better penetration through the cell membrane, resulting in a higher cellular uptake than the conventional macroemulsion. The droplet size is crucial to improve drug penetration in which the smaller size has a better absorption due to the larger interfacial surface area [53]. On the other hand, Shen and colleagues have conducted a study on the cellular uptake of astaxanthin nanoemulsion using six different emulsifiers: Tween20, Whey Protein Isolate (WPI), WPI-lecithin, Polymerized Whey Protein (PWP), PWP-lecithin and lecithin. Among all the formulations, Tween 20-based nanoemulsion had the smallest particle size of 193.87 ± 3.84 nm but did not exhibit the highest astaxanthin Caco-2 cellular uptake. However, WPI-stabilized emulsion with particle size of 203.81 ± 7.27 nm demonstrated the highest cellular intake, approximately 10-fold higher than the free astaxanthin despite not having the smallest droplet size. The authors suggested that whey protein-based carriers were able to deliver astaxanthin in a bioavailable and concentrated form, resulting in higher bioavailability of astaxanthin compared to other emulsifiers. Also, the effect of the digestive system on whey proteins-based emulsions resulted in better absorption of the droplets by the enterocyte cells which also contribute to the increment of astaxanthin bioavailability [54].

In another study, Domínguez-Hernández et al., observed an increase in the bioavailability of astaxanthin in rats that were fed with nano-emulsified astaxanthin prepared using canola oil and Tween 40 at 10 wt.% at surfactants: oil ratios. The formulation showed a 7.5-fold increment in comparison to the reference solution (astaxanthin mixed with canola oil). In this study, the nano-emulsified astaxanthin with size ranging between 1 to 200 nm was produced by employing a low-energy method using a magnetic stirrer at approximately 2000 rpm. It was proven that the smaller particle diameter provides a large interfacial area for the release of the bioactive astaxanthin, hence allowing better absorption that leads to the improvement of astaxanthin bioavailability. The addition of Tween 40 as the emulsifier was also believed to improve the permeability of astaxanthin [55]. This study supported the findings of an earlier study conducted by Affandi and the research team, in which the authors compared the oral bioavailability of astaxanthin from three formulations with different size of particles. In this experiment, they formulated astaxanthin nanoemulsion by mixing astaxanthin oil (16%) with purified water (80% *w*/*w*) and the addition of Tween 80 and lecithin as emulsifiers. The nano-sized emulsion was produced by prolonging the homogenization process for 5 cycles. They noted that the bioavailability of nano-sized astaxanthin emulsion was 1.5 and 2.2 times higher when compared with macro-sized astaxanthin emulsion and the reference formulation, respectively. The mean C_max_ of nano-sized astaxanthin emulsion, macro-sized astaxanthin emulsion and the reference oil were 698.7 ± 38.7 ng/mL, 465.1 ± 43.0 ng/mL and 313.3 ± 12.9 ng/mL, respectively. A significant increment of the C_max_ was correlated to the size reduction of the emulsions; larger surface area produced from the smaller size oil droplet size resulted in higher absorption, better solubility and permeation. The elimination half-life (t_1/2_) of nano-sized astaxanthin emulsion, macro-sized astaxanthin emulsion and the reference oil were 30.1 ± 7.7 h, 29.5 ± 5.4 h and 27.1 ± 6.9 h respectively, indicating that particle size did not have an impact on t_1/2_ [55,56].

Nanoemulsion is one of the best delivery systems for astaxanthin due to its effective solubilization in the hydrophobic core of the lipid droplets owing to the nature of the lipid phase that dissolves lipophilic compounds [57]. Also, the Brownian motion effects dominated the gravitational forces due to its smaller droplet sizes compared to the conventional macroemulsion. Therefore, this system is kinetically stable and the destabilization of nanoemulsions is slow [52]. In addition, the high surface area as a result of small particle size increases the bio-absorption and bioavailability of astaxanthin. As suggested by Haung et al., the more rapid degradation and the quicker release rates in nanoemulsion results in the easier release of astaxanthin into the aqueous gastrointestinal environment from the oil phase due to the large surface area. 

Despite these advantages, there are several events that may occur leading to instability and separation of the nanoemulsions. For example, turbidity of nanoemulsions happens due to flocculation, sedimentation, creaming and cracking. Flocculation occurs when the droplets are closely attached together, forming large clumps; then, the clumps start to settle down (sedimentation) or rise up (creaming) in the emulsion. Usually, the droplets in the emulsion can be redistributed with gentle shaking or agitation. On the other hand, cracking is the irreversible effect of nanoemulsion instability [50]. This happens when there is separation of the dispersed and continuous phase into two individual layers.

#### 4.1.2. Liposomes

Liposomes are either synthetic or natural phospholipids in an aqueous phase composing closed spherical vesicles. There are several methods for preparing liposomes such as the reverse-phase evaporation technique and detergent removal technique [51]. The formation of liposomes is a spontaneous process, as the lipid bilayer closes itself because of the interactions between water molecules and the hydrophobic parts of the phospholipids. Then, the amphiphilic phospholipids self-associated, forming bilayers [58]. Liposomes are highly stable enough to carry active ingredients such as vaccines, and steroids as well as mostly used to deliver lipophilic bioactive like astaxanthin [51,58,59]. The ability to protect the active constituents from degradation and improve drug targeting are the main reasons for liposomes being selected over the conventional dosage form as carrier [44]. The diameter of liposome ranges between 400 nm to 2.5 mm, an appropriate particle size for this delivery system to pass through the endothelial cell membrane by diffusion or lipid-mediated endocytosis [7,58,60].

The bioavailability of astaxanthin-loaded liposomes was assessed by Sangsuriyawong and colleagues through the Caco-2 cellular uptake. They reported that the carriers with higher phospholipid compositions have better adherence to membranes and penetration across intestinal barriers due to lipophilic nature. On top of that, the astaxanthin-loaded liposome with 70% phospholipid composition (PC) was highly absorbed by Caco-2 cells (95.33%), while the formulation with 23% phospholipid composition did not exhibit cellular uptake. This could be due to the smaller size of 70%-PC liposomes (0.14 µm) compared to 23%-PC liposomes with a diameter of 0.31 µm. The lipophilicity and small particle size were agreed by many researchers to be among the important factors for permeation across the intestinal barriers that eventually enhances the bioavailability of active compounds [60].

Although liposomes have exhibited potential in delivering a variety of bioactive compounds, there were several limitations associated with the application of this carrier system that includes rapid leakage of water-soluble drug in the presence of blood components, low encapsulation efficiency and poor stability for storage [44]. Therefore, there is an improvised and closely related spherical vesicle innovation that was postulated to encapsulate drugs better than liposomes, known as niosomes. This newer generation of drug carrier is a non-ionic surfactant-based system that assembles itself into vesicles. Heat and physical agitation were involved during the formation of the closed bilayer vesicles from the non-ionic surfactants, where the hydrophilic portion is in contact with the aqueous solvent, while the hydrophobic portion is facing away from the aqueous portion. There are several forces present inside the vesicles such as van der Waals forces, repulsive forces and entropic repulsive forces that maintain the vesicular structure of the niosomes [61]. The presence of hydrophilic, lipophilic and amphiphilic moieties in the vesicles resulting in unilamellar or multilamellar closed bilayer niosomes. These structures have been demonstrated to have resistance against enzymatic hydrolysis and acid media. Also, the structure allows encapsulation of both water- and lipid-soluble compounds, where the hydrophiles can settle in the vesicular or the bilayer surface aqueous phase while the hydrophobes can be encapsulated in the non-aqueous bilayer core [59,62]. However, to the best of our knowledge, no study has been conducted on the incorporation of astaxanthin into niosomes, to date. This could offer opportunities for future development of niosomes for the delivery of astaxanthin. 

#### 4.1.3. Solid Lipid Nanoparticles (SLNs) and Nanostructured Lipid Carriers (NLCs)

Solid lipid nanoparticles (SLNs) are another type of delivery system that has bloomed in the field of nanomedicine, most recently. SLNs consist of solid biodegradable lipids that are stabilized by surfactants producing colloidal carriers, with a diameter ranging between 50 to 1000 nm [63]. On the other hand, nanostructured lipid carriers (NLCs) are the second generation of lipid nanoparticles after SLNs. NLCs are nanoparticles produced by mixing solid and liquid lipid (oil), creating a different matrix structure when compared to SLNs [39,64]. This lipid composition differentiates the two systems, as SLNs contain a matrix of lipids that do not melt at room and physiological temperature, while NLCs consist of a blend of solid and liquid lipids [65]. Both of the systems can be synthesized using various methods that include high-pressure homogenization and microemulsion [64]. The applications of SLNs and NLCs are wide. Both nanocarriers are being employed in the diagnostic field, food industry and delivery of nutraceuticals. These systems are suitable for the administration of lipophilic drugs that have limited oral absorption [39].

Wang and co-authors conducted a study observing an enhanced antioxidant activity of astaxanthin-loaded SLNs in simulated GI fluids. It was found that astaxanthin incorporated in the SLNs exhibited stronger antioxidant activity even at low concentration of 0.25 µg/mL, compared to the free astaxanthin that only showed activity at 10 µg/mL. They observed the antioxidant activity in the radicals-scavenging assay, ABTS (2,2’-azino-bis(3-ethylbenzothiazoline-6-sulfonic acid)) assay and revealed that free astaxanthin was separated out from the media. The free astaxanthin showed poor solubility, which could be associated with low antioxidant activity [66]. In another in vitro study by Li et al., astaxanthin-loaded SLNs showed promising stability and enhanced release of astaxanthin in the simulated GI juices when compared to the free astaxanthin. The free astaxanthin was rapidly decomposed, up to 68.3 ± 1.5%, while the astaxanthin-loaded SLNs only showed small decomposition percentage of less than 10%, indicating SLNs were capable of protecting astaxanthin from degradation. SLNs also offered better protection for astaxanthin than nanoemulsion, since astaxanthin was embedded in lipid nucleation that underwent recrystallization during the formulation process [67].

SLNs have several advantages for the delivery of astaxanthin such as modifiable release and easy incorporation process. However, there are few drawbacks associated with SLNs which include the inability to control drug expulsion from the carriers during storage. To improve these limitations, NLCs have been developed and utilized for drug delivery systems. The lipid recrystallization for NLCs that is fewer than SLNs, can reduce the expulsion of the active ingredients during storage [68]. Besides, NLCs that are developed from a mixture of solid and liquid lipids were noted to have structural imperfections. The liquid lipids in NLCs provide lesser crystalline arrangement, and the spaces between the crystal lattice can equip higher amounts of drug than the perfect crystalline structure in SLNs, which only offer little space for drugs incorporation [64,69]. The potential of NLCs for oral delivery of astaxanthin have been investigated by a number of researchers. NLCs offer chemical stability between lipophilic compounds and the lipid-based carriers that also have shown good dispersibility and high stability in water-based products compared to SLNs [41]. A study by Mao et al., has proven that astaxanthin-loaded nanostructured lipid carriers had higher antioxidant activity as observed from the DPPH (2,2-diphenyl-1-picryl-hydrazyl-hydrate) assay. They found that the DPPH free radicals scavenging rate for astaxanthin-loaded NLCs and free astaxanthin were at 100% and 94%, respectively. The result indicated that there was almost no loss in antioxidant activity when incorporating astaxanthin into the NLCs. Also, it was found that the particle size did not significantly increase in the stomach condition, indicating astaxanthin-loaded NLCs were not influenced by the high acid conditions and pepsin suggesting, NLC’s suitability as a stable carrier for astaxanthin. However, the PDI was significantly increased in the intestine condition due to the presence of anionic components such as bile salts, phospholipid and free fatty acids. These anionic components, nonetheless, may help in the absorption of astaxanthin, which would lead to the improvement of bioavailability of astaxanthin [70]. 

### 4.2. Other Polymeric Nanoparticles—Chitosan-Based Nanoparticles and Poly(Lactic-Co-Glycolic Acid) (PLGA)

Another type of nano-formulation that has been considered as a suitable carrier for astaxanthin is chitosan-based nanoparticles. Chitosan is the only positively charged polysaccharide and has been employed in various applications such as preparation of colloidal nanoparticles that wields interaction with negatively charged polyelectrolytes electrostatically [71]. Chitosan-based nanoparticles can be produced using various methods such as cross-linking process, ionic gelation and solvent evaporation [71,72,73]. This biopolymer-based formulation has been gaining interest by researchers due to numerous advantages. For example, chitosan-based nanocarriers are biodegradable, safe and have better absorption through the Peyer’s patches [41].

Recently, Q. Hu and colleagues found an innovative approach to enhance the oral bioavailability of astaxanthin by developing complex chitosan-casein-oxidized dextran nanoparticles through a cross-linking process. The nanoparticles with size of 120 nm had an improved dispersibility of astaxanthin in polybutylene succinate (PBS) with better stability in the GI fluids. This property was contributed by the strong covalent bond that formed between the aldehyde group of the oxidized dextran with the amino groups of the stearic acid-chitosan conjugate and sodium caseinate via a Schiff base reaction. Furthermore, the antioxidant activity of astaxanthin-loaded complex chitosan-casein-oxidized dextran nanoparticles could reach up to 85.6% in the ABTS assay as compared to the free astaxanthin. Free astaxanthin showed low antioxidant activity due to its poor dispersibility in PBS that led to limited contact with free radicals. However, these biopolymer-based nanoparticles might be dissociated if administered orally due to the presence of a dextran-splitting enzyme (dextran-1,6-glucosidase) in the liver that breaks down the oxidized dextran before reaching the target [71]. Hence, the innovation of astaxanthin-loaded chitosan-based nanoparticles requires further exploration.

Kim and the research team have developed chitosan-tripolyphosphate nanoparticles via ionic gelation for loading of astaxanthin and studied its stability, antioxidant activity and bioavailability of astaxanthin. They have confirmed that the release of astaxanthin from the nanoparticles in simulated gastric and intestinal fluid was prolonged, indicating stability of the formulation in the GI tract. In vivo study was also conducted using Ferric Reducing Antioxidant Power (FRAP) assay to assess the antioxidant activity of the astaxanthin-loaded chitosan-tripolyphosphate nanoparticles. The result showed a rapid increment of antioxidant activity in the beginning of the test for the free astaxanthin (106.68 ± 17.93 µmol/L), with a slower increase for astaxanthin-loaded nanoparticles (85.33 ± 22.45 µmol/L). After observing the assay for 4 h, FRAP values for astaxanthin-loaded nanoparticles showed a consistent increment, indicating antioxidant activity was constantly executed. On the other hand, the FRAP values for free astaxanthin were reduced over time, which could be due to low solubility of free astaxanthin in the alkaline condition and decreased antioxidant activity of astaxanthin. It was suggested that these drawbacks can be overcome by encapsulating astaxanthin within the chitosan-based nanoparticles to enhance the bioavailability as well as providing a sustained release of oral astaxanthin [72].

In a very recent study, Zhu et al., formulated a novel carrier polyethylene glycol (PEG)-grafted chitosan for encapsulating astaxanthin by employing solvent evaporation method. They found that astaxanthin-encapsulated chitosan was well absorbed (20 µg/mL) in the intestinal, unlike the free astaxanthin. The free astaxanthin did not show a reading due to its insolubility in blank intestinal perfusion fluid containing sodium carboxymethylcellulose that retained astaxanthin in the small intestine. The C_max_ obtained from an in vivo study for astaxanthin-encapsulated in chitosan and free astaxanthin were 2264.03 ± 64.58 ng/mL and 231.45 ± 7.47 ng/mL, respectively [74]. This indicated a greater absorption of astaxanthin for a chitosan-based formulation than the free astaxanthin. Also, the AUC_(0–60)_ for astaxanthin-encapsulated chitosan was significantly higher than the free astaxanthin, by 6.2 times. This result showed a remarkable increment of the bioavailability of astaxanthin when encapsulated into PEG-grafted chitosan [73].

Lipophilic bioactive such as astaxanthin has difficulties being readily infused, even via intraperitoneal injection, due to its poor bioavailability. When astaxanthin was encapsulated into chitosan-based nanoparticles, it resulted in a sustained release profile, protected against GI degradation and improved solubility of astaxanthin in the core. It was also suggested that chitosan-based nanoparticles with sustain-released property are suitable for targeting astaxanthin towards specific sites along with enhancement of the bio-accessibility [59]. On top of that, the nano-sized chitosan-based formulations have enhanced water solubility due to the higher surface area. This factor helps the particles to pass through the cell membranes, contributing to the improvement of oral bioavailability of astaxanthin [72,73]. Overall, encapsulating astaxanthin into chitosan-based nanoparticles weighted more advantages than the disadvantages.

Poly(lactic-co-glycolic acid) (PLGA) is a polymeric nanoparticle that has proven to become one of the promising approaches after being approved pharmaceutically as safe and stable for oral ingestion. It is commonly used as a carrier for active ingredients such as paclitaxel, coenzyme Q10 and vitamin E due to its biodegradability and lower toxicity through hydrolysis in the GI environment [46,74]. PLGA-based nanoparticles for astaxanthin can be developed by employing several methods such as anti-solvent precipitation, nanoprecipitation and emulsion solvent evaporation [46,75,76].

Liu and colleagues designed astaxanthin-loaded core-shell nanoparticles consisting of chitosan oligosaccharides and PLGA as an approach to enhance the solubility and bioavailability of astaxanthin. The nanoparticles were produced by employing anti-solvent precipitation method for the core and electrostatic deposition method for the chitosan oligosaccharides coating. The authors reported that the astaxanthin-coated nanoparticles showed the fastest release rate in an acidic environment of the in vitro GI tract simulation. In the beginning of the experiment, an initial burst release was observed due to rapid diffusion of astaxanthin located around the surface of the nanoparticles. Then, the remaining astaxanthin encapsulated in the core of the nanoparticles slowly diffused through the core-shell structure to allow more sustained release in both stomach (pH 2.1) and small intestine (pH 7.4) conditions. They also found that the nanoparticles remained in their original color with no segregation, indicating a stable formulation when stored at room temperature for up to 72 h. The chitosan oligosaccharide-coated astaxanthin-PLGA showed good dispersibility in water, which helped in its solubility improvement, hence exhibiting its potential as carrier for enhancement of astaxanthin oral bioavailability [45].

## 5. Limitations of Astaxanthin-Loaded Nanoparticles

Recently, it is suggested that the oral bioavailability of astaxanthin can be improved with nano-formulations. A number of in vitro and in vivo studies were reviewed to discover the effectiveness of formulating astaxanthin into nanoparticles, and their pharmacokinetic profiles are reported for the upcoming innovations. Astaxanthin has been incorporated into nanoemulsions, liposomes, SLN, chitosan-based nanoparticles and other polymeric nanoparticles. These newly developed formulations are proven to improve cell permeability, cell absorption and enhance the oral bioavailability of astaxanthin with no toxicity being reported thus far. However, there are challenges for the application and commercialization of astaxanthin nano-formulations, whereby a very limited number of scientific and clinical studies were reported to date. This includes the toxicity profile of astaxanthin-loaded nano-formulations, which is still scarce and the current findings are highly dependent on the in vitro studies [77]. Therefore, further scientific and clinical studies are warranted to provide better insights of astaxanthin-loaded nanoparticles in terms of its application, therapeutics effects and toxicity profile. Regulatory actions for astaxanthin-loaded nanoparticles should also be taken into measures as nano-formulations have been growing widely in the research industry [77]. Moreover, with ongoing innovations of the nano-formulations of astaxanthin, future studies are able to establish a suitable delivery system and their uses in nutraceutical or pharmaceutical purposes. In addition, prospective supplementations of astaxanthin nano-formulations for critical diseases such as cancer could also be proposed with the insights of its pharmacotherapeutic, pharmacokinetics and toxicity profile.

## 6. Conclusions

Despite having promising pharmacological properties, the current research data of astaxanthin reported for its clinical applications are still scarce. There were very limited studies reporting the oral bioavailability of astaxanthin, thus far. Also, the toxicity profile of astaxanthin nano-formulations were not widely studied and the current findings were highly dependent on the in vitro studies. Therefore, with ongoing innovations of the nano-formulations of astaxanthin, it is strongly suggested that future clinical studies could establish pharmacokinetic and toxicity profiles of the suitable delivery systems and their therapeutic uses in nutraceutical or pharmaceutical purposes. In addition, prospective supplementations of astaxanthin nano-formulations for critical diseases such as cancer could also be proposed with the insights of its pharmacotherapeutic, pharmacokinetics and toxicity profile.

In conclusion, the pharmacological activities of astaxanthin have led to a wide range of therapeutics effects. Regardless of the promising outcomes, astaxanthin is not well utilized due to lipophilicity of the compound that limits the dissolution in the gastrointestinal fluids and causes reduced bioavailability. Moreover, astaxanthin is not synthesized by humans; this makes it essential to be taken as an exogenous supplement, prompting a suitable nano-formulation of astaxanthin to be developed in the near future to achieve better dissolution, enhanced bioavailability and improved therapeutic applications. Therefore, the pharmacokinetic and therapeutic profile of astaxanthin nano-formulations are beneficial for future commercialization and its therapeutic applications for human wellness. 

## Figures and Tables

**Table 1:** Pharmacokinetic parameters of astaxanthin.

Author(s)	Year	Factors Affecting Astaxanthin Bioavailability	Study Size, *n*	AUC_(0–∞)_ ± S.D. (µg.h.l^−1^)		Summary of Findings
Yumika Okada, Masaharu Ishikura & Takashi Maoka	2009	Oral bioavailability of astaxanthin based on two different timings	20	Before-meal group	2996 ± 969	The AUC_(0–∞)_ for the after-meal group was 2.5 times higher than the before-meal group. This may be due to the additional lipid substances that enhance the absorption of astaxanthin.
				After-meal group	7526 ± 3300	
Johanna Mercke Odeberg, Ake Lignell, Annette Pettersson & Peter Höglund	2003	Oral bioavailability of astaxanthin in four different formulations	32	Reference ^1^	1347 ± 501	Formulation B had the highest AUC_(0–∞)_ among all four formulations. This may be due to the proportion of lipids in the formulation that improve the absorption of astaxanthin.
				A ^2^	2216 ± 574 *	
				B ^3^	4960 ± 1504 *	
				C ^4^	2580 ± 850 *	
Yumika Okada, Masaharu Ishikura & Takashi Maoka	2009	The impact smoking on oral bioavailability of astaxanthin	20	Non-smokers group	7526 ± 3300	The smoking habit affected the pharmacokinetics parameters; AUC_(0–∞)_ was 1.15 times higher in the non-smokers group compared to smokers’ group and 1.65 times for t_1/2_. This may be due to the high oxidative stress led to high oxidation of astaxanthin.
				Smokers group	6518 ± 4125	

^1^ Reference (Algal meat, algal fat & dextrin), ^2^ A (Algal meat, algal fat, palm oil & Polysorbate 80 (hydrophilic)), ^3^ B (Algal meat, algal fat, glycerol mono- and dioleate & Polysorbate 80 (hydrophilic)) and ^4^ C (Algal meat, algal fat, glycerol mono- and dioleate, polysorbate 80 & sorbitan monooleate 80). * *p* < 0.05, statistically significant difference compared to the reference.

**Table 2:** Summary findings of astaxanthin nano-formulations.

Nano-Formulations	Nanoparticle Size	Method of Preparation	In Vitro	In Vivo	Findings	Reference
Nanoemulsions	145.6 ± 27.7 to 155.0 ± 40.8 nm	Self-emulsification	✓	✓	Increase permeability through Caco-2 cells by 80% Prove to cure lung metastatic melanoma in vivo	[53]
	193.87 ± 3.84 to 286.52 ± 19.75 nm	High shear homogenization, ultrasonication	✓		Different emulsifiers resulted in varying mean droplet sizes WPI-stabilized emulsion reported to have the highest Caco-2 cells intake	[54]
	0.023 ± 3.83 to 19.65 ± 3.34 nm	Self-emulsification, high pressure homogenization, ultrasonication	✓	✓	Nano-emulsification has improved the permeability of astaxanthin in rats The particle size of the emulsion influenced the absorption and permeability of astaxanthin	[55]
	0.128 ± 0.015 µm	Homogenization		✓	Nanosized emulsion was prepared by prolonging the homogenization process from the macro-sized emulsion Nanosized emulsion has the highest C_max_ and AUC_(0−∞)_ compared to macro-sized emulsion and reference astaxanthin	[56]
Liposomes	0.14 ± 0.01 nm	Thin-film hydration	✓		Different composition of phospholipid content (23% and 70%) produced different particle size 70%-PC liposomes were absorbed at 95.33% while there was no absorption for 23%-PC liposomes	[61]
SLNs	150 to 800 nm	Solvent-free homogenization, sonication	✓		The optimized liposomes improved the GI stability Astaxanthin-encapsulated liposomes showed 70% encapsulation efficiency	[67]
Chitosan-based	91.7 to 148.8 nm	Ionic gelation crosslinker-free fabrication method	✓		Nanoparticles have improved the dispersibility of astaxanthin in PBS assay Astaxanthin-loaded chitosan based nanoparticles showed 85.6% antioxidant activity in the ABTS assay	[72]
	483.9 ± 148.4 to 653.8 ± 215.1 nm	Ionic gelation	✓	✓	The astaxanthin-loaded chitosan-tripolyphosphate nanoparticles prolonged the release of astaxanthin in simulated GI and intestinal fluids In FRAP assay, the astaxanthin-loaded chitosan-tripolyphosphate nanoparticles exhibited consistent antioxidant activities compared to free astaxanthin	[73]
	122.1 ± 6.4 nm	Solvent evaporation	✓	✓	PEG-grafted chitosan loaded with astaxanthin showed absorption, whereas the free astaxanthin could not In animal model, the astaxanthin-loaded chitosan nanoparticles showed significantly high C_max_ by 9.8 times compared to the free astaxanthin	[74]
PLGA-based	150 nm	Anti-solvent precipitation, electrostatic deposition	✓		Astaxanthin-coated PLGA nanoparticles showed the fastest release rate in the simulated GI tract condition. Astaxanthin-nanoparticles also showed good dispersibility in water indicating good solubility No segregation and color changes happened indicating a stable formulation	[46]

Notes: Symbol: ✓ indicates the study setting(s) in which the corresponding astaxanthin nano-formulations were tested. Abbreviations: SLNs, solid lipid nanoparticles; WPI, whey protein isolate; C_max_, peak plasma concentration; AUC, area under the curve; PC, phospholipid content; GI, gastrointestinal; PBS, polybutylene succinate; ABTS, 2,2’-azino-bis(3-ethylbenzothiazoline-6-sulfonic acid); FRAP, ferric reducing antioxidant power; PEG, polyethylene glycol; PLGA, poly(lactic-co-glycolic acid).

## Abbreviations

UV, ultraviolet; USFDA, United States Food and Drug Administration; DNA, deoxynucleic acid; ROS, reactive oxygen species; HO-1, heme oxygenase-1; Nrf2, nuclear factor erythroid 2-related factor; GPX1, glutathione peroxidase 1; SOD2, superoxide dismutase 2; SOD, superoxide dismutase; HDF, human dermal fibroblasts; NRK, normal rat kidney epithelial cells; Con A, concanavalin A; LPS, lipopolysaccharide; LOOH, lipid hydroperoxide; LDL, low density lipoprotein; V-70, 2, 2’-azobis(4-methoxy-2, 4-dimethyl-valeronitrile); SHR, spontaneously hypertensive rats; SHR-SP, spontaneously hypertensive rats-stroke prone; NADPH, nicotinamide adenine dinucleotide phosphate; XO, xanthine oxidase; NOx, nitrogen oxides; AdipoR1, adiponectin receptor 1; AdipoR2, adiponectin receptor 2; t2dm, type 2 diabetes mellitus; GI, gastrointestinal; HDL, high density lipoprotein; C_max_, peak plasma concentration; T_max_, time taken to achieve peak plasma concentration; AUC_0-__∞_, area under the curve; t_1/2_, half-life; Caco-2 cells, human colon cancer cell line; TAP-nanoemulsion, tocopheryl polyethylene glycol succinate-nanoemulsion; WPI, whey protein isolate; PWP, polymerized whey protein; PC, phospholipid composition; SLN, solid lipid nanoparticle; NLC, nanostructured lipid carrier; ABTS, 2,2’-azino-bis(3-ethylbenzothiazoline-6-sulfonic acid); DPPH, (2,2-diphenyl-1-picryl-hydrazyl-hydrate); PDI, polydispersity index; PLGA, poly(lactic-co-glycolic acid); PBS, polybutylene succinate; FRAP, ferric reducing antioxidant power; PEG, polyethylene glycol.
